# Efficacy and safety of Trastuzumab Emtansine in treating human epidermal growth factor receptor 2-positive metastatic breast cancer in Chinese population: a real-world multicenter study

**DOI:** 10.3389/fmed.2024.1383279

**Published:** 2024-04-29

**Authors:** Miao He, Wen Zhao, Peng Wang, Wenhuan Li, Hanhan Chen, Zonghuai Yuan, Guangye Pan, Hong Gao, Lijun Sun, Jiahui Chu, Li Li, Yu Hu

**Affiliations:** ^1^Department of Medical Oncology, Qilu Hospital, Cheeloo College of Medicine, Shandong University, Jinan, Shandong Province, China; ^2^Department of Medical Oncology, Qilu Hospital (Qingdao), Cheeloo College of Medicine, Shandong University, Qingdao, China; ^3^Department of Medical Oncology, Qingdao Shibei Changqing Hospital, Qingdao, Shandong Province, China; ^4^Department of Chemotherapy, Shandong Provincial Hospital Affiliated to Shandong First Medical University, Jinan, Shandong Province, China; ^5^Department of Breast and Thyroid Surgery, Affiliated Hospital of Shandong University of Traditional Chinese Medicine, Jinan, China; ^6^Department of General Surgery, People’s Hospital of Rizhao, Rizhao, Shandong Province, China; ^7^Department of Breast and Thyroid Surgery, Rizhao Traditional Chinese Medical Hospital, Rizhao, Shandong Province, China; ^8^Department of Breast and Thyroid Surgery, People’s Hospital of Juxian, Rizhao, Shandong Province, China; ^9^Department of Pharmacy, Qilu Hospital, Cheeloo College of Medicine, Shandong University, Jinan, Shandong Province, China

**Keywords:** trastuzumab emtansine, antibody–drug conjugate, T-DM1, DCR, platelet

## Abstract

**Background:**

Trastuzumab emtansine (T-DM1) has been approved worldwide for treating metastatic breast cancer (mBC) in patients who have received first-line therapy, shown disease progression, and are human epidermal growth factor receptor 2 (HER2)-positive. T-DM1 received approval in China to treat early-stage breast cancer (BC) in 2020 and for mBC in 2021. In March 2023, T-DM1 was included in medical insurance coverage, significantly expanding the eligible population.

**Materials and methods:**

This post-marketing observational study aimed to assess the safety and effectiveness of T-DM1 in real-world clinical practice in China. This study enrolled 31 individuals with HER2-positive early-stage BC and 70 individuals with HER2-positive advanced BC from 8 study centers in Shandong Province, China. The T-DM1 dosage was 3.6 mg/kg injected intravenously every 3 weeks until the disease advanced or the drug toxicity became uncontrollable, whichever occurred earlier. Additionally, efficacy and safety information on T-DM1 were collected.

**Results:**

During the 7-month follow-up period, no recurrence or metastases were observed in patients who had early-stage BC. The disease control rate was 31.43% (22/70) in patients with advanced BC. The most common adverse effect of T-DM1 was thrombocytopenia, with an incidence of 69.31% (70/101), and the probability of Grade ≥ 3 thrombocytopenia was 11.88% (12/101).

**Conclusion:**

This real-world study demonstrated that T-DM1 had good efficacy and was well tolerated by both HER2-positive early-stage BC and mBC patients.

## 1 Introduction

Breast cancer (BC) is a common malignancy among women worldwide, and 70–80% of patients with early-stage non-metastatic disease can potentially be cured. According to current treatments, advanced BC with distant metastases is generally considered to have a poor prognosis (1). BC is highly heterogeneous, and treatment strategies vary according to the molecular characteristics, including human epidermal growth factor receptor 2 (HER2) activation, expression of hormone receptors, gene mutations, and immune microenvironment markers (2).

Approximately 20% of BC cases over-expressing HER2 (3) have a poorer prognosis and short overall survival (OS) (4). In the last two decades, various HER2-targeted therapies have been developed, including humanized monoclonal antibodies such as trastuzumab (5) or pertuzumab (6), as well as tyrosine kinase inhibitors (TKIs) such as lapatinib (7) or neratinib (8). Currently, the most authoritative treatment for patients with metastatic disease includes the “dual HER-2 blockade” therapy using trastuzumab and pertuzumab plus paclitaxel (9). This treatment regimen improves progression-free survival (PFS) and OS. Nevertheless, despite these achievements, HER2-positive metastatic BC (mBC) remains incurable.

The introduction of trastuzumab has significantly affected the prognosis of individuals with HER2-positive BC and influenced its diagnosis and treatment approaches. It represents a significant breakthrough in the drug treatment of BC (10, 11). The combination of trastuzumab with other chemotherapeutic agents improves the prognosis of patients with metastatic diseases and decreases cancer recurrence (12–14). Despite the superior efficacy of trastuzumab, most patients with advanced disease develop resistance to this drug; therefore, there remains an ongoing requirement to discover new drugs that specifically target progressive HER2-amplified diseases (15). HER2-directed therapy works best when combined with cytotoxic chemotherapy; hence, a novel antibody–drug conjugate (ADC) has been developed (15).

Trastuzumab emtansine (T-DM1) comprises trastuzumab and the tubulin inhibitor, DM1, which are bound via a stable, non-lysable, non-reducing thioether linker. DM1 can prevent the assembly of mitotic functional spindles by effectively binding to tubulin, thus depolymerizing it, and can induce cell cycle arrest and apoptosis. T-DM1 possesses the same mechanism of action as trastuzumab, which effectively blocks the HER2 signaling pathway (16). The 2012 EMILIA study provided evidence for the effectiveness of T-DM1 as a second-line treatment for HER2-positive mBC (17). T-DM1 was approved by the Food and Drug Administration in the following year as a standalone therapy for treating mBC (18). In 2019, the KATHERINE study laid the groundwork for using T-DM1 to manage early-stage BC (19). This study investigated the effectiveness and safety of T-DM1 in real-world clinical practice, specifically for treating early-stage or advanced HER2-positive BC in China.

## 2 Materials and methods

### 2.1 Study population

This Phase 4, multicentric, observational, clinical study was conducted in eight sites in the Shandong Province of China from 1 March 2023 to 28 June 2023. Our research was approved by the Medical Ethical Committee of Qilu Hospital of Shandong University. All participants signed a comprehensive informed consent document, which included the purpose and procedures of the study. The study was conducted according to the principles of Good Clinical Practice.

This study had specific inclusion criteria. It included individuals who demonstrated HER2 overexpression with an immunohistochemistry (IHC) score of 3+. Alternatively, those with a 2+ IHC score and positive results in fluorescence *in situ* hybridization testing were also considered eligible for participation. Patients with pathologically confirmed unilateral, measurable invasive mBC who received chemotherapy, specifically trastuzumab and a taxane drug were included. Patients also comprised those with non-invasive primary BC who had completed at least six cycles of paclitaxel adjuvant chemotherapy, as well as those with residual invasive diseases detected in the surgical specimens of breast or axillary lymph nodes. Other eligibility criteria included an Eastern Cooperative Oncology Group performance status of 0 or 1, successful recuperation from any treatment-related toxicities, left ventricular ejection fraction (LVEF) ≥50%, absolute neutrophil count ≥1,500 cells/mm^3^, hemoglobin ≥90 g/L, platelet count ≥100,000 cells/mm^3^, aspartate aminotransferase and alanine aminotransferase ≤2.5 × upper limits of normal (ULN), and total bilirubin ≤1.5 × ULN.

The exclusion criteria included anti-HER2 ADC therapy, chemotherapy, hormone therapy, radiotherapy, or BC surgery within 3 weeks before the screening process; a history of symptomatic chronic heart failure or treatment for severe arrhythmia; severe systemic disease; HIV or hepatitis B infection; pregnancy or breastfeeding; allergy to T-DM1; and any other medical or psychiatric condition deemed unsuitable for the study by the investigator.

### 2.2 Treatment methods

T-DM1 administration involved an intravenous infusion of 3.6 mg/kg over 90 min during the initial cycle. Subsequently, a dose of 3.6 mg/kg was administered over 30 min every 3 weeks until the investigator detected uncontrolled toxicity or disease progression, whichever occurred earlier. Uncontrolled toxicity refers to a serious treatment-related adverse event (AE) that prevents the continued use of a therapeutic drug. Before each infusion, premedication was administered, which included analgesics/antipyretics and antihistamines. The study allowed the use of other supportive medications, such as ondansetron or palonosetron, as well as palliative care during the study.

### 2.3 Efficacy and toxicity

According to the RECIST 1.1 (20) guidelines, tumor response was evaluated after 6 weeks of treatment or earlier if clear signs of disease progression appeared rapidly. The primary outcomes were disease control rate (DCR) and disease-free survival (DFS). DCR generally refers to the percentage of cases with remission and stable lesions in the number of evaluable cases after treatment. The DCR was calculated by summing the rates of complete response (CR), partial response (PR), and stable disease (SD). CR means that the patient has been treated for the tumor in multiple ways, that the majority of the lesions have disappeared, that no new lesions have formed, and that the tumor marker examination has continued normally for more than 4 weeks. PR means that the maximum size reduction of the target lesion is ≥30% and maintained for at least 4 weeks. SD means that the sum of the large diameter of the patient’s target lesions does not shrink by more than 30%, or the increase does not exceed 20%. DFS refers to the time from a clinically proven CR to local recurrence or distant metastasis.

Toxicities were evaluated and categorized as per the National Cancer Institute Common Toxicity Criteria, version 5.0. The deadline for data collection for the study was 5 June 2023.

### 2.4 Statistical analysis

We used chi-square tests or Fisher’s exact test to assess the difference in the efficacy of T-DM1 between early-stage and advanced BC patients. Statistical analyses were performed using Statistical Product and Service Solutions 24.0. GraphPad Prism was used to plot the Kaplan–Meier survival curves. The log-rank test was used to analyze the PFS. Results with a *p*-value of ≤0.05 were considered statistically significant.

## 3 Results

### 3.1 Patient information

This study included 101 women with HER2-positive B, and T-DM1 treatment was administered. The median age was 51 years (range, 21–74). Hormone receptor positivity was observed in 44 cases (43.56%). Distant metastasis was reported in 70 cases (69.31%), including 38 (54.29%) with visceral metastasis and 10 (26.32%) with brain metastasis. The baseline characteristics are shown in Table 1. All patients were previously treated with paclitaxel and trastuzumab.

**Table 1:** Baseline clinicopathological and disease characteristics of 101 human epidermal growth factor receptor 2-positive breast cancer patients.

Characteristics	Total (N/%)
Median age(range), years	51(21–74)
21–45 years	27 (26.73%)
46–60 years	56 (55.45%)
>60 years	18 (17.82%)
ECOG performance status	
0–1	101 (100%)
≥2	0 (0%)
Menopausal status	
Premenopausal	41 (40.59%)
Postmenopausal	60 (59.41%)
Hormone-receptor status	
−	57 (56.44%)
+	44 (43.56%)
Lines of T-DM1 treatment in mBC	
1	0 (0%)
2	7 (10.00%)
≥3	63 (90.00%)
Previous usage of trastuzumab	
Yes	101 (100%)
No	0 (0%)
Distant metastasis	
No	31 (30.69%)
Yes	70 (69.31%)
Visceral metastasis	
No	32 (45.71%)
Yes	38 (54.29%)
Number of visceral metastasis sites	
1	11 (28.95%)
≥2	27 (71.05%)
Brain metastasis	
Yes	10 (26.32%)
No	28 (73.68%)

ECOG, Eastern Cooperative Oncology Group; mBC, metastatic breast cancer.

### 3.2 Treatment administration

Table 2 summarizes the previous treatment methods.

**Table 2:** Previous treatment for all patients.

Previous treatment		Early stage (*N* = 31)	Proportion (%)	Advanced (*N* = 70)	Proportion (%)
Radiotherapy		8	25.81	19	27.14
Endocrine therapy		8	25.81	12	17.14
Previous use of chemotherapeutic drugs	Paclitaxel	31	100.00	70	100.00
	Anthracycline	0	0	14	20.00
	Cyclophosphamide	0	0	17	24.29
	Pyrimidines	0	0	18	25.71
	Vinorelbine	0	0	11	15.71
	Platinum	21	67.74	8	11.43
Previous use of Targeted therapy	Trastuzumab	31	100.00	70	100.00
	Pertuzumab	22	70.97	13	18.57
	Pyrotinib	0	0	19	27.14
	Lapatinib	0	0	0	0
	Inetetamab	0	0	7	10.00
Methotrexate		0	0	2	2.86
CDK4/6 inhibitor		0	0	1	1.43

### 3.3 Clinical efficacy

**Figure 1:**
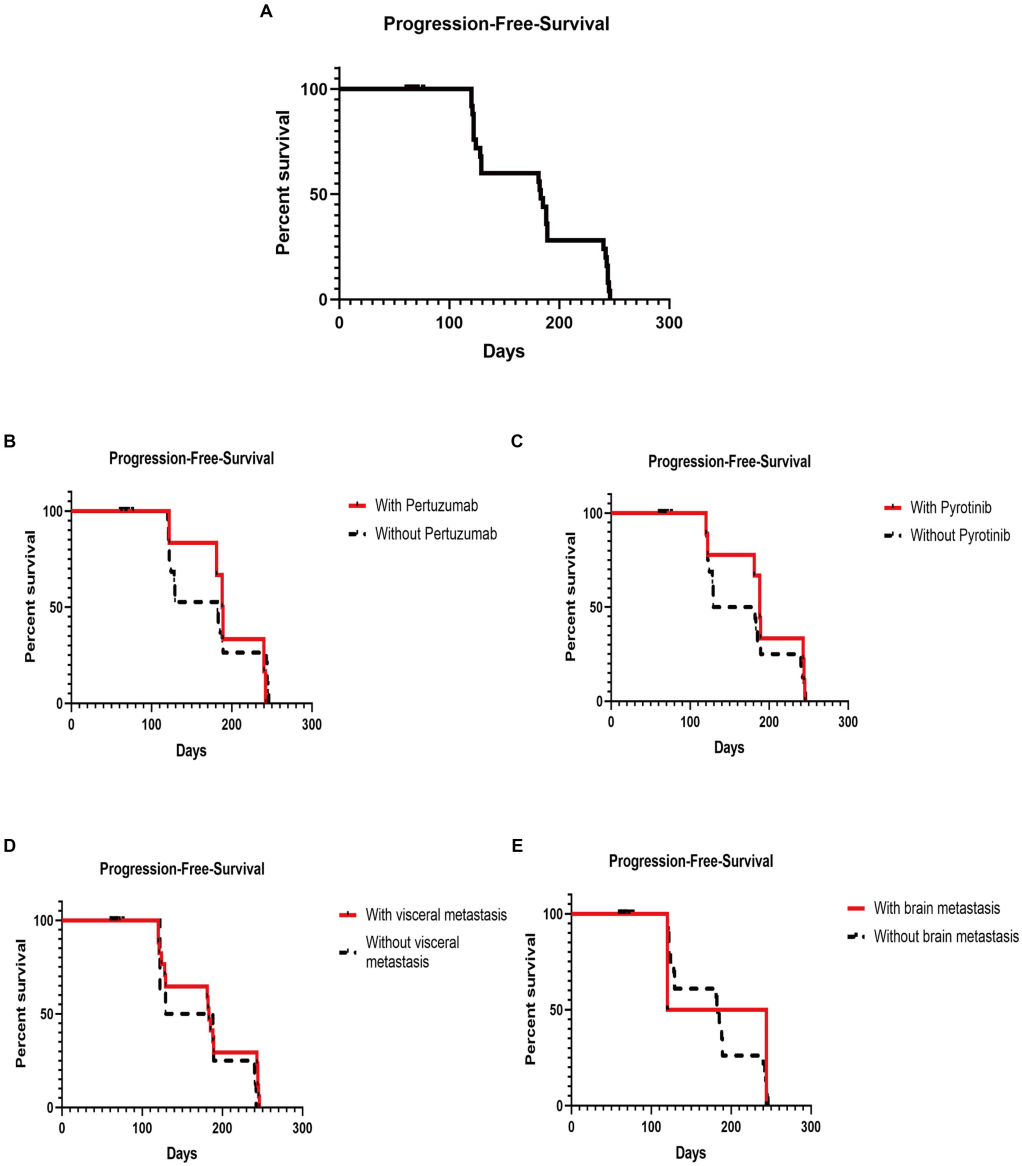
Kaplan–Meier plot of progression-free survival and log-rank analysis of the predictors of trastuzumab emtansine treatment. **(A)** Kaplan–Meier plot of the PFS of all patients subjected to T-DM1 treatment; **(B)** Kaplan–Meier plot of the PFS of patients with and without previous pertuzumab treatment; **(C)** Kaplan–Meier plot of the PFS of patients with and without exposure to pyrotinib; **(D)** Kaplan–Meier plot of the PFS of patients with and without visceral metastasis; **(E)** Kaplan–Meier plot of the PFS of patients with and without brain metastasis (PFS, progression-free survival; T-DM1, trastuzumab emtansine).

The efficacy in patients with diverse clinicopathologic and disease features until the cutoff date is shown in Table 3. Among the patients with advanced BC disease, the DCR did not differ significantly between those with different clinicopathologic and disease characteristics treated with T-DM1 (Figure 1A). The DCR in patients with advanced disease was 31.43% (22/70). However, the median PFS (mPFS) was longer in patients who had used pertuzumab (188 days; 95% CI, 178.40–197.60) than in those who had not (182 days; 95% CI, 123.35–240.66), without a statistically significant difference (*p* = 0.92; Figure 1B). Similarly, the mPFS was longer in patients who had used pyrotinib (188 days; 95% CI, 177.77–198.23) than in those who had not (129 days; 95% CI, 58.44–199.56), without a statistically significant difference (*p* = 0.49; Figure 1C). Furthermore, the mPFS was longer in patients with visceral metastases (183 days; 95% CI, 177.62–188.38) than in those without visceral metastases (129 days; 95% CI, 37.53–220.47), without a statistically significant difference (*p* = 0.38; Figure 1D). The mPFS of patients without brain metastases (183 days; 95% CI, 176.74–189.26) was longer than that of patients with brain metastases (120 days), without a statistically significant difference (*p* = 0.84; Figure 1E). Until the cutoff date, no patients with early-stage BC who were followed up for 7 months showed disease progression after T-DM1 treatment.

**Table 3:** Evaluation of efficacy in patients with different clinicopathologic and disease characteristics.

Characteristics	Early stage (*N* = 31) (N/%)	No recurrence or metastasis	DFS	Advanced (*N* = 70) (N/%)	CR + PR + SD	DCR	*p*
Sex							
Male	00 (0%)	0	0	0 (0%)	0	0	−
Female	31 (100%)	31	100%	70 (100%)	22	31.43%	
ECOG performance status							
0–1	31 (100%)	31	100%	70 (100%)	22	31.43%	−
≥2	0 (0%)	0	0	0 (0%)	0	0%	
Menopausal status							0.93
Premenopausal	14 (45.16%)	14	100%	26 (37.14%)	8	30.77%	
Postmenopausal	17 (54.84%)	17	100%	44 (62.86%)	14	31.82%	
Tumor histology							
Ductal	26 (83.87%)	26	100%	58 (82.86%)	20	34.48%	0.39
Other	5 (16.13%)	5	100%	12 (17.14%)	2	16.67%	
Hormone receptor status							
−	14 (45.16%)	14	100%	31 (44.29%)	10	32.26%	0.89
+	17 (54.84%)	17	100%	39 (55.71%)	12	30.77%	
Ki-67							
<14	8 (25.81%)	8	100%	10 (14.29%)	2	20.00%	0.64
≥14	23 (74.19%)	23	100%	60 (85.71%)	20	33.33%	
Surgical Histopathological grade							
≤2	21 (67.74%)	21	100%	35 (50.00%)	9	25.71%	0.30
>2	10 (32.26%)	10	100%	35 (50.00%)	13	37.14%	
Number of lymph nodes							
≤3	25 (80.65%)	25	100%	47 (67.14%)	17	36.17%	0.22
>3	6 (19.35%)	6	100%	23 (32.86%)	5	21.74%	
Surgical Tumor Tissue							
Size							
≤2 cm	11 (35.48%)	11	100%	36 (51.43%)	11	30.56%	0.87
>2 cm	20 (64.52%)	20	100%	34 (48.57%)	11	32.35%	
Visceral Metastasis							
No	−	−	−	32 (45.71%)	13	40.63%	0.13
Yes	−	−	−	38 (54.29%)	9	23.68%	
Number of Visceral Metastasis							
1–2		−	−	11 (28.95%)	6	54.55%	0.40
≥3	−	−	−	27 (71.05%)	9	33.33%	
Lines of T-DM1 treatment in MBC							
1	−	−	−	0 (0%)	0	0	
2	−	−	−	7 (10.00%)	3	42.86%	0.63
≥3	−	−	−	63 (90.00%)	19	30.16%	

CR, complete response; PR, partial response; SD, stable disease; ECOG, Eastern Cooperative Oncology Group; mBC, metastatic breast cancer.

### 3.4 Safety

We evaluated 101 patients for toxicity. The overall toxicity rate was 70% (49/70) in the advanced BC group and 71% (22/31) in the early-stage BC group.

Platelet counts decreased in 70% (49/70) of patients in the advanced BC group and 67.74% (21/31) of patients in the early-stage BC group. Patients with platelets <50*10^9/L accounted for 4.29% (3/70) and 29.03% (9/31) of the advanced BC and early-stage BC groups, respectively. The following AEs were commonly reported in association with treatment: diarrhea (1/101, 0.99%), neutropenia (55/101, 54.46%), and nausea and vomiting (16/101, 15.84%). Hematological toxicity and gastrointestinal toxicity were the most common. Details of AE occurrences are shown in Table 4. These AEs are generally tolerated and manageable. AEs can be improved to Grade 1 or 2 after therapy with recombinant human thrombopoietin injection and/or aprepitant, ondansetron, and others, without the interruption of the T-DM1 therapy. Two patients in the early-stage BC group experienced a reduction in the platelet count, leading to a dose reduction of T-DM1. Additionally, one patient in the advanced BC group had their T-DM1 dose reduced due to nausea and diarrhea.

**Table 4:** Trastuzumab emtansine-related adverse events of all grades.

Grading of adverse events	Early stage (*N* = 31) (N/%)	Advanced (*N* = 70)(N/%)
Leukopenia -	9 (29.03%)	21 (30.00%)
1–2	20 (64.52%)	48 (68.57%)
3–4	2 (6.45%)	1 (1.43%)
Neutropenia		
−	11 (35.48%)	35 (50.00%)
1–2	18 (58.06%)	34 (48.57%)
3–4	2 (6.45%)	1 (1.43%)
Anemia		
−	14 (45.16%)	40 (57.14%)
1–2	17 (54.84%)	29 (41.43%)
3–4	0	1 (1.43%)
Thrombocytopenia**-**	10 (32.26%)	21 (30.00%)
1–2	12 (38.71%)	46 (65.71%)
3–4	9 (29.03%)	3 (4.29%)
Abnormal liver function		
−	31 (100%)	69 (98.57%)
+	0 (0%)	1 (1.43%)
Diarrhea		
−	31 (100%)	69 (98.57%)
+	0 (0%)	1 (1.43%)
Nausea		
−	29 (40.59%)	60 (85.71%)
+	2 (59.41%)	10 (14.29%)
Vomit		
−	30 (96.77%)	67 (95.71%)
+	1 (3.23%)	3 (4.29%)
Hand-foot syndrome		
−	31 (100%)	70 (100%)
+	0 (0%)	0 (0%)
Rash		
−	31 (100%)	70 (100%)
+	0 (0%)	0 (0%)
Acroparesthesia		
−	30 (96.77%)	70 (100%)
+	1 (3.23%)	0 (0%)
LVEF decreases		
−	31 (100%)	70 (100%)
+	0 (0%)	0 (0%)
Upper respiratory or urinary tract infection		
−	31 (100%)	68 (97.14%)
+	0 (100%)	2 (2.86%)

LVEF, left ventricular ejection fraction.

## 4 Discussion

The major outcomes of this study demonstrate that T-DM1 exhibits favorable efficacy in patients with early-stage HER2-positive BC, which corroborates the findings of previous clinical trials (19, 21). The DCR is not ideal in advanced BC, which could be due to the administration of T-DM1 as the second-line therapy. The decrease in the platelet count was most significant in terms of the safety of T-DM1 administration (22). The DCR was higher for hormone receptor-negative HER2-positive mBC than for hormone receptor-positive HER2-positive mBC (33.33% versus 28.21%) without a statistically significant difference between these two subgroups.

T-DM1 treatment showed a greater survival benefit in patients who had previously used pertuzumab or pyrotinib compared to those who had not. A trend toward better outcomes with T-DM1 was observed in patients with visceral metastases but no brain metastases as compared to those without metastases. However, no statistical difference was observed in these results, which could be associated with the fact that the average number of treatment lines for advanced BC treated with T-DM1 was more than five. Key evidence supporting continuous HER2 inhibition after trastuzumab treatment has been reported in BC patients. BC patients showing disease progression after treatment with trastuzumab and anthracyclines or taxanes were included in the EGF100151 study (23). In a randomized study, patients were assigned to receive lapatinib plus capecitabine or capecitabine monotherapy. The PFS of the group receiving combination treatment was 8.4 months. In the GBG26 study, the addition of trastuzumab to capecitabine extended the mPFS to 8.2 months (24). Other trials have also demonstrated the importance of continued anti-HER2 therapy (25–27). The T⁃DM1 drug comprises a combination of the targeted therapeutic drug trastuzumab and the cytotoxic drug DM1. The targeting effect of trastuzumab and the anti-tumor effect of DM1 are exerted simultaneously, and the anti-tumor drugs are delivered to the target cells for a better therapeutic effect (28–32). The Emilia trial showed that the PFS of the T-DM1 group could be nearly 9.6 months following the failure of trastuzumab (22). In our study, the mPFS of patients with disease progression after trastuzumab and/or TKIs has not been determined.

The EMILIA study further demonstrated that T-DM1 not only exhibited improved efficacy but also a favorable safety profile. Specifically, the TDM-1 group showed a decreased incidence of Grade ≥ 3 severe AEs, with thrombocytopenia being the most commonly reported (22). Furthermore, the TH3RESA clinical trial was conducted to compare the efficacy of T-DM1 therapy with physician choice therapy (TPC). The study aimed to evaluate the efficacy of T-DM1 in patients who had undergone prior treatment with a minimum of two HER2-targeted therapies, such as lapatinib and trastuzumab (33). Most patients had metastatic disease and had previously undergone at least four prior treatments, and most patients in the TPC group had been treated with a regimen containing trastuzumab. A significant improvement in PFS was observed with T-DM1 compared to TPC (mPFS, 6.2 months versus 3.3 months). In the last analysis of OS, patients treated with T-DM1 showed a significantly longer median OS than that of the control group (median OS, 22.7 months versus 15.8 months). Consistent with the findings of the EMILIA study, thrombocytopenia was identified as a more common AE associated with T-DM1 treatment (34). In the TDM4450g study, patients with HER2-positive mBC or recurrent locally advanced BC were assigned to receive either T-DM1 or trastuzumab plus docetaxel as the first-line treatment. T-DM1 has a better safety profile and fewer serious AEs compared to other treatment regimens (35).

Additionally, the KATHERINE trial study validated T-DM1 for treating residual aggressive HER2-positive BC. In the study, 5.7% of the patients developed thrombocytopenia (19, 36). The efficacy of T-DM1 has also been demonstrated in other clinical trials (37–51). For example, T-DM1 was active and well tolerated in a population with brain metastases from BC, and metronomic temozolomide in combination with standard dose T-DM1 has shown low-grade toxicity and potential activity in the secondary prevention of HER2+ brain metastases (41, 52), Moreover, thrombocytopenia has been a dose-limiting side effect that can lead to treatment discontinuation (53, 54).

Regarding the treatment safety in this study, the most common Grade ≥ 3 AE among the people analyzed in Shandong Province, China, was peripheral thrombocytopenia. Satisfactory relief could be achieved after administering thrombopoietin to stimulate platelet production, and most patients could achieve a good platelet count tolerance in subsequent courses. Other common AEs related to T-DM1 are nausea, elevated transaminase, diarrhea, and vomiting, most of which were of Grade 1–2 severity. These findings align with those of previous studies (30, 36). Therefore, the AEs of T-DM1 in the real world are tolerable and easily controlled (55). Notably, thrombocytopenia can be caused by impaired platelet production as well as reduced platelet survival in circulation (56). T-DM1 can inhibit the production of megakaryocyte platelets (57–59). T-DM1 is absorbed by megakaryocytes, which inhibits megakaryocyte differentiation, disrupts platelet formation by inducing abnormal tubulin organization, and inhibits microtubule dynamic instability. However, clinical studies have also shown that the platelet survival rate of patients treated with T-DM1 has a statistically significant and gradual downward trend, and T-DM1 directly decreases the patients’ platelet circulation time and function (60).

Our data also included patients with Grade 4 thrombocytopenia. We examined the genes of two patients with very low platelet counts, and both revealed mutation sites, namely *MDR121* exon G2677 homozygous mutation, *CYP3A4* heterozygous mutation, and *CYP3A5* heterozygous mutation. Several studies have found that there are two molecular “efflux pumps” in tumor cell membranes, namely P-glycoprotein (P-gp) and multi-drug resistance-associated protein. These pumps are responsible for expelling therapeutic drugs from the cancer cells, leading to the phenomenon called multi-drug resistance (61, 62). Among them, P-gp is the transporter of the adenosine triphosphate-binding cassette, encoded by the *ABCB1/MDR1* gene (63, 64). Multi-mutation analysis revealed that *MDR1* had a large genetic variation, indicating that single-nucleotide polymorphisms (SNPs) may significantly affect the expression and function of P-gp transporters (65–67). Of the SNPs reported in the *MDR1* gene, *G2677T/A* in exon 21 has been extensively studied and determined to be of functional significance. There are racial differences in the localization of this gene region (68–70).The *G2677T/A* SNP of exon 21 leads to 893Ser (G2677T) and the much rarer 893Thr (G2677A), which can alter transporter function or expression (71, 72). Furthermore, *G2677* has prognostic significance in disease progression (73). A particular study revealed a strong association between the *G2677T/A* SNP and the efficacy of paclitaxel treatment in patients with ovarian cancer. Patients with pure mutations had a high probability of responding to paclitaxel therapy. The frequency of the T or A allele was also higher in the group of patients with a better prognosis than in those with a poor prognosis (74). Moreover, several studies have shown that the *MDR1* genotype is strongly associated with the efficacy and toxicity of chemotherapy in BC (75, 76). For example, adriamycin is affected by the ABCB1 transporter (72, 77). *CYP3A4* and *CYP3A5* are members of the human cytochrome P450 gene family, located on chromosome 7, and primarily involved in the metabolism of drugs. They break down drugs into molecules that can be absorbed by major tissues. Genetic abnormalities affect the activity of the gene-encoded protein, which in turn affects the metabolism of the drug in the human body, leading to too high or too low drug concentration in the blood, which results in a too high or too low therapeutic effect and even drug side effects. For example, cyclophosphamide is an active drug activated by a variety of cytochrome P450 enzymes, including *CYP3A5* (78). These enzyme and transporter genes all have genetic variations to some degree, characterized by SNPs. In summary, we hypothesize that T-DM1 and its active metabolites are substrates for *MDR1*-encoded glycoprotein (P-gp). Furthermore, the liver enzymes CYP3A4 and CYP3A5 are also trapped in the drug metabolism of T-DM1, and patients with mutations in these two genes are susceptible to AEs associated with T-DM1, especially thrombocytopenia.

The findings of this real-world study corroborated the data from the EMILIA study and the KATHERINE study. The mechanism of thrombocytopenia revealed the mutation site of the metabolic enzyme.

This retrospective study has some limitations. First, the sample size was relatively small, which may have introduced a bias in the results. Second, the questionnaire survey and telephonic follow-up for AE analysis could introduce subjectivity. Third, the follow-up period was relatively short. Therefore, further validation of these findings is warranted through large prospective cohort studies.

## 5 Conclusion

The main AE of T-DM1 as the second-line therapy for HER2-positive mBC was thrombocytopenia in the Chinese population. It was overall well tolerated and exhibited promising anti-tumor activity, even in patients in whom previous trastuzumab and pertuzumab therapy had failed.

## Data availability statement

The raw data supporting the conclusions of this article will be made available by the authors, without undue reservation.

## Ethics statement

The studies involving humans were approved by Medical Ethical Committee of Qilu Hospital of Shandong University. The studies were conducted in accordance with the local legislation and institutional requirements. The ethics committee/institutional review board waived the requirement of written informed consent for participation from the participants or the participants’ legal guardians/next of kin because The treatments used are guideline-based.

## Author contributions

MH: Formal analysis, Writing – original draft. WZ: Writing – review & editing. PW: Data curation, Writing – review & editing. WL: Data curation, Writing – review & editing. HC: Data curation, Writing – review & editing. ZY: Data curation, Writing – review & editing. GP: Data curation, Writing – review & editing. HG: Data curation, Writing – review & editing. LS: Data curation, Writing – review & editing. JC: Data curation, Writing – review & editing. LL: Visualization, Writing – review & editing. YH: Conceptualization, Methodology, Writing – review & editing.
